# P-2238. A Novel Transcriptomic-based Host Response Point-of-care Test to Distinguish Bacterial from Viral Infection: Results of a Diagnostic Accuracy Study in Adults Hospitalised with Acute Respiratory Illness

**DOI:** 10.1093/ofid/ofae631.2391

**Published:** 2025-01-29

**Authors:** Bilge Eylem Dedeoglu, Alex R Tanner, Nathan J Brendish, Helen Moyses, Tristan William Clark

**Affiliations:** Oxford University Hospitals NHS Foundation Trust, Oxford, England, United Kingdom; University Hospital Southampton NHS Foundation Trust, Southampton, England, United Kingdom; University of Southampton & University Hospitals Southampton NHS Trust, Southampton, England, United Kingdom; University Hospital Southampton NHS Foundation Trust, Southampton, England, United Kingdom; University of Southampton, Southampton, England, United Kingdom

## Abstract

**Background:**

Acute respiratory infections (ARI) are the commonest reason for antibiotic use, but much of this is unnecessary as a high proportion is caused by viruses. Distinguishing bacterial from viral ARIs is challenging, and detecting viral pathogens does not rule out concurrent bacterial infection, leading to inappropriate antibiotic use and driving antimicrobial resistance. Novel tests assessing the host immune response have the potential to differentiate bacterial and viral ARIs and direct antibiotic use. In this study, we evaluated the accuracy of the TriVerity Acute Infection and Sepsis Test (Inflammatix, Sunnyvale, CA, US), a transcriptomic-based host response point-of-care test which measures 29 mRNAs to determine the likelihood of bacterial and viral infection, in adults with acute respiratory illness.

**Figure:**
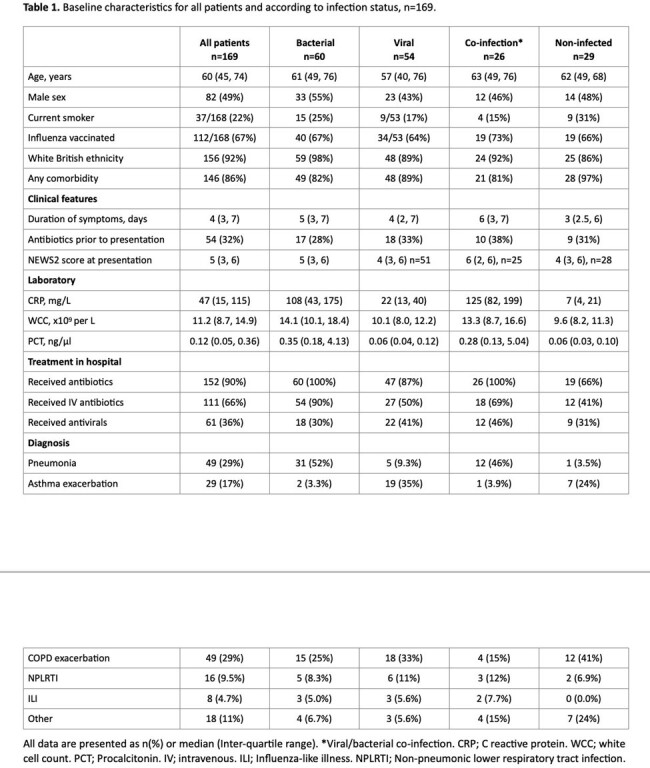

**Methods:**

Adults hospitalised with ARI were enrolled as part of the FluPOC study. The aetiology for each patient (bacterial and viral), was clinically adjudicated by 3 clinicians independently using a pre-specified guidance document. RNA paxgene blood samples were tested using the TriVerity test on the Myrna platform (Inflammatix, Sunnyvale CA, US). The test assigns one of five probability bands (very low, low, moderate, high, very high) for both bacterial and viral infection. Measures of diagnostic accuracy were calculated between the TriVerity results and adjudicated infection status.

**Figure:**
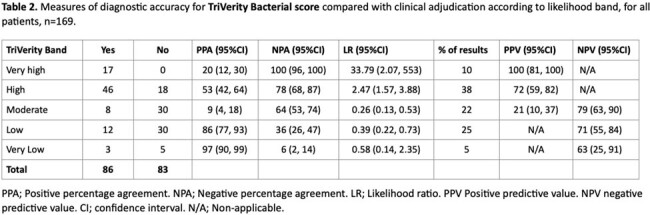

**Results:**

169 patients were tested, and TriVerity testing gave an initial valid result in 168 (99%). The median (IQR) time to result was 32.49 (32.41, 32.53) minutes. Median age was 60 (45, 74) years and baseline characteristics are shown in table 1. 61 (36%) were adjudicated as bacterial, 54 (32%) as viral, 26 (15%) as viral/bacterial co-infection and 29 (17%) as non-infected. Measures of diagnostic accuracy for bacterial and viral infection compared to adjudication are shown in table 2 and 3 respectively.

**Figure:**
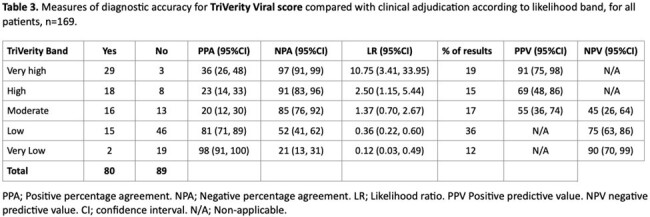

**Conclusion:**

The TriVerity test demonstrated good levels of accuracy in distinguishing bacterial from viral ARIs compared with clinical adjudication and was reliable with a short turnaround time. TriVerity could support early informed antibiotic decisions and reduce unnecessary antibiotics use. Prospective impact trials with clinical outcome measures are needed.

**Disclosures:**

Tristan William Clark, BM MRCP DTM&H MD, Biomerieux/Biofire diagnositcs: Advisor/Consultant|Biomerieux/Biofire diagnositcs: Grant/Research Support|Biomerieux/Biofire diagnositcs: Honoraria|Cepheid: Advisor/Consultant|GSK: Advisor/Consultant|Inflammatix: Grant/Research Support|Roche: Advisor/Consultant|Sanofi: Advisor/Consultant|Sanofi: Grant/Research Support|Sequirus: Advisor/Consultant|Sherlock Biosciences: Grant/Research Support|Shionogi: Advisor/Consultant|Synairgen: Advisor/Consultant|Synairgen: Grant/Research Support|Synairgen: Honoraria|Synairgen: Stocks/Bonds (Public Company)

